# Cochlear Implantation in Patients with Keratitis-Ichthyosis-Deafness Syndrome: A Report of Two Cases

**DOI:** 10.1155/2017/3913187

**Published:** 2017-10-02

**Authors:** Birgul Gumus, Armagan Incesulu, Mehmet Ozgur Pinarbasli

**Affiliations:** Department of Otorhinolaryngology, Faculty of Medicine, Eskişehir Osmangazi University, Eskişehir, Turkey

## Abstract

**Background:**

Keratitis-ichthyosis-deafness (KID) syndrome is a syndrome which presents with hearing loss and visual and keratinization disorders. In such patients, hearing aids cannot be effectively used in the rehabilitation of hearing loss because of the frequent blockage of the external ear canal with epithelial debris and due to dry and tense skin of the external ear canal. Moreover, severe or profound hearing loss also limits the benefits gained from the conventional hearing aids. On the other hand, cochlear implantation is a method that has been used in limited cases in the literature.

**Case Report:**

This study presents the results of cochlear implantation applied in our clinic to two children who had been diagnosed with KID. Audiological assessments before and after the cochlear implant operation were performed using pure-tone audiometry, immittance audiometry, and auditory brainstem response (ABR), and the postoperative follow-up was conducted using pure-tone audiometry.

**Conclusion:**

Skin problems, visual disturbances, and other additional problems complicate the short-term and long-term rehabilitation after implantation in individuals with KID syndrome. Close monitoring should be exercised due to possible skin complications that may develop during the postoperative period. The families and rehabilitation teams should be warned about the possible visual disturbances and skin complications.

## 1. Introduction

Keratitis-ichthyosis-deafness (KID) syndrome is a rare congenital disorder characterized by keratitis, congenital ichthyosis, and deafness. KID is inherited by an autosomal dominant transmission, with a mutation present in the Connexin-26 gene, which is a structural protein [1–4]. Visual problems may be congenital or may develop during early adolescence in patients with KID syndrome. Visual problems are corneal keratitis, sensitivity to bright light, decreased visual acuity, and hyperkeratosis on the eyelid margin [1, 5]. Although it has been demonstrated that hearing loss can be moderate to profound in terms of severity in KID syndrome, it is mostly seen in the form of congenital, severe or profound, and nonprogressive hearing loss. While hearing loss is in the form of sensorineural type, conduction pathologies due to external otitis and otitis media can also be seen [6]. Conventional hearing aids have been used in the rehabilitation of sensorineural hearing losses in individuals with KID syndrome. However, the use of conventional hearing aids may be ineffective due to skin problems and accumulation of debris in the external ear canal [1]. Moreover, since most patients have severe hearing loss, the contribution of conventional hearing loss to speech development may be limited, and in such cases, cochlear implantation is the only option among the current technological advancements.

This study presents the results of cochlear implantation applied to two children with KID syndrome. Before the operation, a proclamation form was obtained from these patients' families.

## 2. Case Report

*Case 1*. The patient was born as a term male infant in the year of 2006. The diagnosis of hearing loss was made at the age of 4 since routine newborn hearing screening was not widely practiced across the country during the period he was born in. Moreover, rehabilitation of his other problems had had priority. After the diagnosis, he started to use a bilateral conventional hearing aid. However, his family stated that he could not use the hearing aid regularly because of debris due to the syndrome and extreme dryness of the skin in the external ear canal. After starting to use the hearing aid, auditory training had been added to the physical rehabilitation he had been using due to his gait problem. His language development had been limited despite the training, and he was referred to our clinic for evaluation with respect to cochlear implantation when he was 7 years old. When the patient came to our clinic, he underwent tests showing severe sensorineural hearing loss bilaterally. The auditory brainstem response (ABR) test was performed preoperatively and the tests did not reveal wave V and cochlear microphonic even in the bilateral maximal stimulation. In the Acoustic Immittance, bilateral Type A tympanogram was obtained but bilateral, ipsilateral, and contralateral reflexes could not be obtained. No bilateral emission response was observed. Based on the electrophysiological results, the bilateral severe sensorineural hearing loss was diagnosed. Free-field pure-tone audiometry was performed to test the benefits of the hearing aids. At 90 dB SPL, no response could be obtained with the hearing aid at any frequency. At the same magnitude level, no response with the hearing aid to speech stimulus was observed.

Based on the assessment by using AGTE (Ankara Development Screening Inventory) by the Department of Pediatric Psychiatry, his development with respect to social skills and self-care was found at the level of 20 months, and his general development was found to be well behind his age. The ophthalmological assessment did not reveal any keratitis findings associated with the syndrome. The preoperative auditory integration assessment using MUSS, MAIS, CAPS, SIR, and Auditory Questionnaire revealed that he used gesture and gabbled in his daily communication.

Cochlear implant surgery was performed in May 2014. A wide postauricular incision was performed and skin was elevated posteriorly. The periost was elevated anteriorly. A place was prepared in the bone for the body of the cochlear implant, and the body was fixed in the hole properly in order to prevent bump formation. The cochlear implantation was performed using the trans-mastoid-facial recess approach. A Cochlear® CI422 Slim Straight Electrode was inserted into the right ear. The intraoperative measurements showed that the impedances of all electrodes were within normal limits. Intraoperative Neural Response Telemetry (NRT) was obtained from the five electrodes* (22, 16, 11, 6, 1)*. No complications were observed related to the cochlear implantation during the 21-month postoperative follow-up period.

**Figure 1:**
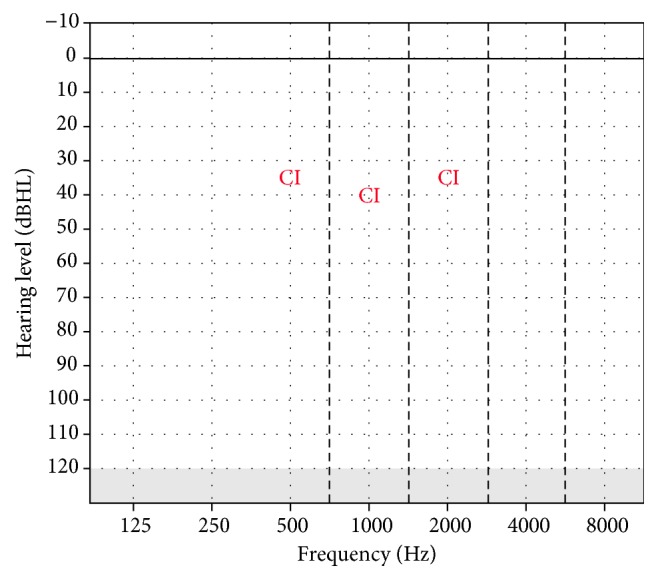
Hearing thresholds with CI in the 3rd month for Case 1.

**Figure 2:**
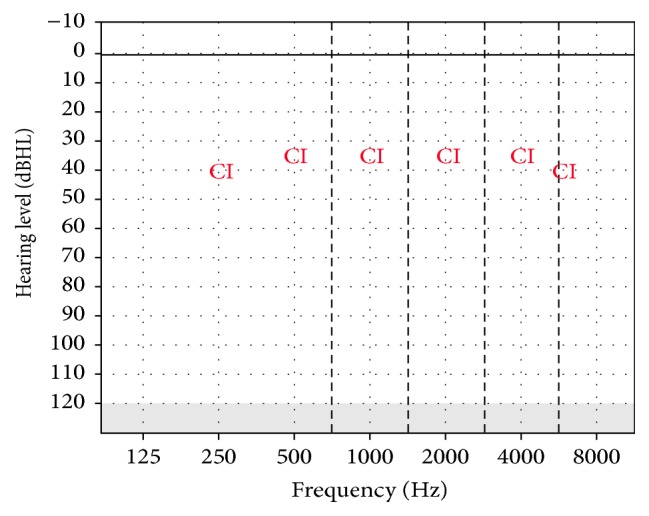
Hearing thresholds with CI in the 1st year for Case 1.

After waiting for completion of the wound healing process for 1 month after the surgery taking into consideration the skin structure associated with the syndrome, the external part of the cochlear implant was placed. Fitting of cochlear implantation was made by using objective and subjective methods. For this purpose, NRT thresholds obtained from the nine* (22, 19, 16, 13, 11, 8, 6, 3, 1)* electrodes have been used for the basal programming. After that, it was controlled whether the patient had any discomfort with live sound. No problem was observed and parents were informed about the details of the cochlear implant. Regular auditory training was continued in his town. He returned to our clinic for the first 3 of 4 routine scheduled appointments during the first postoperative year but could not come for the fourth due to his travel problem. Thereafter, annual appointments were scheduled. No test could be performed for the first auditory check after the fitting of the cochlear implant because of the poor cooperation of the child. On the third postoperative month, thresholds with the cochlear implant were obtained at the frequencies shown in Figure 1, although very-low- and very-high-frequency hearing thresholds could not be obtained. Hearing thresholds were obtained at all frequencies in the first postoperative year. The results are shown in Figure 2. The tests to assess his auditory integration were repeated during the postoperative period. The preoperative and postoperative results are shown in Table 1.

The patient had 8 intelligible words at the end of the first postoperative year and had very good adoption of the device. His family stated that they are very satisfied with the cochlear implantation.

*Case 2*. The patient was born as a term female infant in the year of 2008. Hearing loss was diagnosed during the routine newborn hearing screening at the age of 10 months, and thereafter she had started to use a bilateral conventional hearing aid. Her medical history obtained from the family revealed that she could not use the hearing aid regularly due to skin problems and could not receive regular auditory training since she could not go out of the house due to her seasonal and sun sensitivity. This condition had been accompanied by motor growth retardation, and she was admitted to our clinic for cochlear implantation when she was 5 years old. When the patient came to our clinic, she underwent tests showing severe sensorineural hearing loss bilaterally.

Preoperative ABR test did not reveal wave V and cochlear microphonic in both ears with the maximal stimulation. In the Acoustic Immittance, bilateral Type A tympanogram was obtained but bilateral, ipsilateral, and contralateral reflexes could not be detected. No bilateral otoacoustic emission response was observed. Based on the electrophysiological testing results, bilateral severe sensorineural hearing loss was diagnosed. The hearing aid threshold is at 90 dB SPL, and she had no response to the warble tone stimulus at any frequency. No response to speech stimulus was observed with the hearing aid. These results demonstrated that the patient could not get benefit from the hearing aid.

Based on the AGTE performed by the Department of Pediatric Psychiatry, the general development and the other development areas were well behind her age. All development areas (general development, language-cognitive development, and fine and gross motor development) were found to be well below 30%. The preoperative auditory assessment revealed that her auditory integration-development and language skills were limited. It was observed that she was using gesture and gabbling during the communication. The ophthalmological assessment did not reveal any visual problem.

Cochlear implant surgery was performed with the same surgical steps like in the previous patient in May 2014. A wide postauricular incision was performed and skin was elevated posteriorly. The periost was elevated anteriorly. A place was prepared in the bone for the body of the cochlear implant, and the body was fixed in the space properly in order to prevent bump formation. The cochlear implantation was performed using the trans-mastoid-facial recess approach. A Cochlear CI422 Slim Straight Electrode was inserted into the right ear. The intraoperative measurements showed that the impedances of all electrodes were within normal limits. Intraoperative NRT response was obtained from the five electrodes* (22, 16, 11, 6, 1)*. No complications were observed related to the cochlear implant surgery during the 20 months of the postoperative period.

Taking into consideration the skin structure associated with the syndrome, the external part of the cochlear implant was placed one month later and the first programming was performed. The second fitting was performed one month later. Thereafter, she could not return for the third fitting because of the pregnancy of her mother for the second child and hospitalization of the patient due to frequent epistaxis attacks. She could return for follow-up in the sixth postoperative month, and her family stated that she could not use the cochlear implant regularly because of her hospitalization history. She could return for the last annual follow-up and after the placement of the external part of the cochlear implant. Unfortunately, the patient could not attend the auditory training due to her seasonal sensitivity.

**Figure 3:**
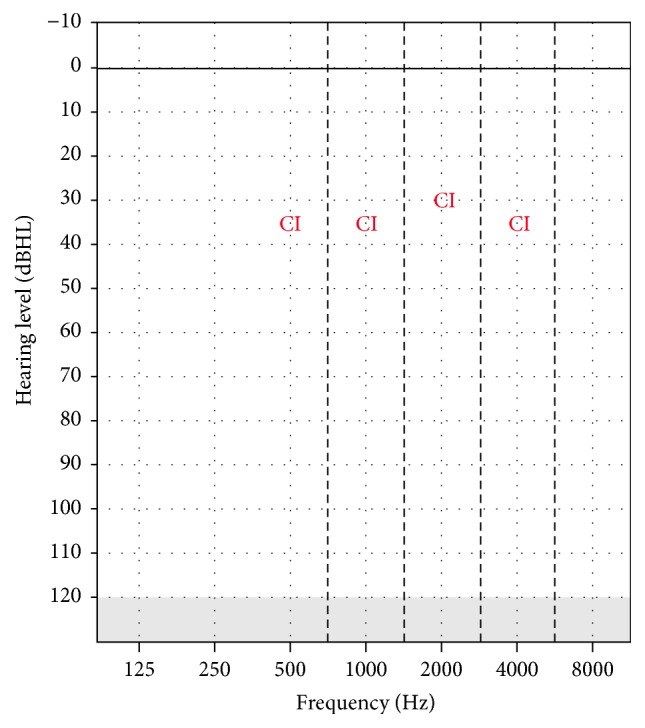
Hearing thresholds with CI in the 1st year for Case 2.

During the fitting of the cochlear implant, all the impedances of the electrodes were checked at all settings and were found to be within normal limits. All programming of the device was made based on the NRT response obtained from the nine electrodes* (22, 19, 16, 13, 11, 8, 6, 3, 1).* Hearing thresholds were attempted to be obtained after each fitting session but could not be obtained, because the patient does not come to regular follow-ups. The hearing thresholds obtained with the cochlear implant on the 1st year of cochlear implant use are shown in Figure 3. The tests to assess her auditory development were repeated during the postoperative period after the 6th month and 1st year when the cochlear implant was adjusted. The preoperative and postoperative auditory integration results are summarized in Table 2.

Her family stated that they are very satisfied with the cochlear implantation despite the mentioned downsides.

## 3. Discussion

Hearing loss is a congenital, often severe or profound condition and this sensorineural deficiency is an important marker in the diagnosis of KID [6]. Our patients had also bilateral, profound, and sensorineural hearing loss. Although the information about the hearing loss being congenital in Case 1 was obtained from the statements of the parents, newborn hearing screening test documented the congenital hearing loss in Case 2.

These patients were referred to us with a KID diagnosis from another hospital. The diagnosis of the disease was made histologically. We wanted to perform a genetic analysis in these patients. However, we did not get these results, because these patients' families did not provide consent to carry out genetic analyses.

In patients with KID syndrome, conduction pathologies may also present due to external ear problems associated with the skin structure in addition to the sensorineural hearing loss [1, 2, 6]. The preferred method for auditory rehabilitation in such patients is conventional hearing aids. However, the thick, dry, and scaly skin complicates the tolerability of hearing aids in the ear canal. Even if we tried to insert the hearing aid, the skin bleeds and then becomes infected. Additionally, skin debris occludes parts of the hearing aid, thereby reducing the sound quality of the hearing aid, or even causing it to fail. Frequent repair of the device will cause the patient to go without acoustic stimuli during the rehabilitation period. The parents of our patients reported that their children could not use their hearing aids regularly because of these problems. The severity of hearing loss is another factor that limits the utility of hearing aids in KID syndrome. In cases of severe or profound hearing loss, the speech sounds cannot be perceived at a sufficient level in some cases even if a hearing aid is used. These patients have profound sensorineural hearing loss so we did not use BAHA. These patients often have otitis externa attacks. Medical treatment should be applied during these attacks.

There are several studies in the literature emphasizing that severe or profound hearing loss requires the use of cochlear implants in order to enhance the speech perception and speech intelligibility [7, 8].

For individuals with hearing loss, lip-reading is important in daily communication even if a hearing aid is used. In KID syndrome, visual problems may be congenital but may also develop with advanced age, thereby complicating lip-reading in this patient population. Cochlear implants should be considered early in the rehabilitation of hearing loss, taking into account factors such as the facts that the use of hearing aids is limited due to skin problems, that hearing aids cannot help the patients in the development of speech due to the severity of hearing loss, and that visual problems that exist or may occur in the future will complicate lip-reading. Also, bilateral implants can be considered in these patients, but in Turkey, the bilateral implant device is not paid for by the state.

There are only a limited number of studies about the cochlear implant application in patients with KID syndrome. Barker and Briggs reported that they applied cochlear implantation to three children with KID syndrome, one unilateral and two bilateral. They found that all three benefited from cochlear implants based on the speech tests during the follow-up periods of 42 months, 26 months, and 21 months, respectively [1]. Hampton et al. [5] reported that an 8-year-old girl developed infection following implantation, and she benefited from the cochlear implant despite the postoperative complications after the revision surgery. Choung et al. [2] reported that implants are a very beneficial method for auditory rehabilitation in this group of patients. Arndt et al. [3] reported that they applied implants to two patients; in one of them, the implant was removed on the fourth postoperative year due to necrosis and another cochlear implant was applied to the other ear, and that cochlear implant contributed to speech development in both patients. Neither of our patients had complications due to cochlear implants during the follow-up periods.

The scores of the questionnaires and scales to determine the development of auditory integration following cochlear implantation were found to generally increase or remain the same during the follow-up periods. MUSS is a questionnaire which is designed to assess the child's use of speech in different natural situations [9]. It was found that MUSS score decreased only in Case 2. In Case 2, the parents stated that she used the cochlear implant only for a few hours or sometimes she did not put on the external part, during the child's hospitalization due to her additional problems. She could not receive training and socialize after the surgery due to her seasonal sensitivity. Unfortunately, the family member could not adequately take care of her. We believe that the low scores found in this questionnaire resulted from these problems. Despite the low scores of MUSS, the parents stated that they are satisfied with the cochlear implantation and the child has become much calmer. In Case 1, auditory training and greater exposure to speech stimuli provided an advantage for the development of speech despite seasonal sensitivity. When the hearing thresholds with a cochlear implant are considered, the results from both cases are at the expected levels with respect to the success of cochlear implants.

Another important issue is the surgical incision to be used in these patients. The minimal incision may be preferred in these patients because of skin and flap problems. However, it may be difficult to place the receiver after a minimal incision. For this reason, we preferred a slightly longer incision and we easily placed the receiver into the bed.

In conclusion, there are difficulties in the use of hearing aids for auditory rehabilitation of individuals with KID syndrome. Taking into account that such patients may have visual problems, the cochlear implant should be considered as an appropriate method for auditory rehabilitation. However, unlike the routine cases, special attention should be paid to certain issues in cochlear implantation. The complication rate in surgery is higher. Therefore, in surgery preparation of flaps, selection of incisions and wound site monitoring are important. Like in our cases, patients may not be able to receive regular rehabilitation due to the syndrome. Therefore, an organization of home training programs and provision of training to the families come into prominence for such patients. Moreover, delayed diagnosis and rehabilitation of hearing loss due to additional problems associated with the syndrome may change the success expected to be achieved after cochlear implantation. It is important that families are informed at each stage and have realistic expectations.

## Figures and Tables

**Table 1:** Auditory Questionnaire results for Case 1.

Assessment time	MAIS	MUSS	CAP	SIR	Auditory Questionnaire
Preoperative	1	3	0	1	0
3rd month	12	8	1	2	1
1st year	19	14	3	2	13

**Table 2:** Auditory integration test results for Case 2.

Assessment time	MAIS	MUSS	CAP	SIR	Auditory Questionnaire
Preoperative	5	19	1	1	0
6th month	18	13	1	1	0
1st year	19	10	3	1	3
